# Sellar and parasellar abnormalities

**DOI:** 10.1590/0100-3984.2018.51.1e3

**Published:** 2018

**Authors:** Gustavo Novelino Simão

**Affiliations:** 1 Attending Physician in the Neuroradiology Department, University of São Paulo Medical School (FMRP-USP), Radiologist at Cedirp, Ribeirão Preto, SP, Brazil. E-mail: gustavonsimao@gmail.com

The sellar and parasellar regions constitute an anatomically complex area comprising various important neurovascular structures within a small space. The sellar region includes the sella turcica and the pituitary gland, together with the ventral adenohypophysis and dorsal neurohypophysis. The parasellar region encompasses the cavernous sinuses, suprasellar cistern, hypothalamus, and ventral inferior third ventricle. Anatomic localization is essential in the creation of a differential diagnosis between sellar and parasellar lesions. The sellar and parasellar regions can be involved in neoplastic, inflammatory/granulomatous, infectious, and vascular diseases, any of which can arise from the pituitary gland, infundibular stalk, hypothalamus, cranial nerves, vascular structures, leptomeninges, or skull base^1^. There are more than 30 processes that can involve the sellar or parasellar region, the entities most commonly seen in general practice including macroadenoma, microadenoma, empty sella, craniopharyngioma, hypothalamicchiasmatic glioma, and meningioma^1^.

Radiologic imaging of the pituitary gland and the parasellar region is challenging because of the small size of the pituitary gland and its close proximity to many important structures. With its high contrast, spatial resolution, and multiplanar capabilities, magnetic resonance imaging (MRI) is the modality of choice to study various diseases of the central nervous system^2^^-^^5^ and can be diagnostic if a process originates from the sellar or parasellar region, as well as characterizing its regional spread. Various MRI sequences have proven to be robust tools for tissue characterization and can determine whether a mass is solid, cystic, hemorrhagic, or fatty, which narrows the differential diagnosis, depending on the location. A standard protocol for MRI of the pituitary gland and parasellar region consists of thin-section (2-3 mm) sagittal and coronal T1-weighted images with and without contrast enhancement. Thin-section T2-weighted imaging can be supplemented to look for cystic lesions. In addition, one T2-weighted scan covering the entire brain should be performed^6^. For some indications, such as the detection of microadenoma, dynamic contrast-enhanced imaging of the pituitary gland should be obtained^1^. Computed tomography (CT) continues to play a role in the evaluation of bone structures, because it can delineate osseous erosion with great detail and characterize calcified tumor matrices^7^.

Interpretative imaging strategies for sellar and parasellar lesions are required to make an accurate differential diagnosis. Hess and Dillon^8^ include some key considerations on that front: determining the normal imaging appearance of the gland and infundibulum, in terms of size and enhancement pattern; localizing the abnormalities as entirely intrasellar, sellar and suprasellar, or entirely suprasellar; characterizing the lesions as entirely solid, entirely cystic, or mixed solid and cystic; categorizing the lesion margins as circumscribed or invasive; distinguishing imaging features that are unique or highly suggestive of cysts, low T2 signal intensity, calcification, or fluid-fluid levels; and identification of mass effect on the optic apparatus, invasion of the cavernous sinuses, and abnormalities located elsewhere in the brain.

The article authored by Eduardo et al.^9^ and published in this issue of Radiologia Brasileira makes a significant contribution to the understanding of sellar and parasellar abnormalities. The authors provide an overview of the most relevant MRI and CT characteristics of pituitary tumors, as well as congenital, vascular, inflammatory, and infectious lesions, found in the sellar/parasellar region, in order to increase the accuracy of the differential diagnosis.

## References

[1] Chin BM, Orlandi RR, Wiggins 3rd RH (2012). Evaluation of the sellar and parasellar regions. Magn Reson Imaging Clin N Am.

[2] Alves LLF, Martino MS, Ortiz Sobrinho C (2017). Brain changes on magnetic resonance imaging in school-age
children who had been preterm infants with intracranial
hemorrhage. Radiol Bras.

[3] Niemeyer B, Muniz BC, Gasparetto EL (2017). Congenital Zika syndrome and neuroimaging findings: what do we
know so far?. Radiol Bras.

[4] Duarte SBL, Oshima MM, Mesquita JVA (2017). Magnetic resonance imaging findings in central nervous system
cryptococcosis: comparison between immunocompetent and immunocompromised
patients. Radiol Bras.

[5] Niemeyer B, Correia RS, Antunes LO (2017). The diagnostic challenge of dizziness: computed tomography and
magnetic resonance imaging findings. Radiol Bras.

[6] Rennert J, Doerfler A (2007). Imaging of sellar and parasellar lesions. Clin Neurol Neurosurg.

[7] Zamora C, Castillo M (2017). Sellar and parasellar imaging. Neurosurgery.

[8] Hess CP, Dillon WP (2012). Imaging the pituitary and parasellar region. Neurosurg Clin N Am.

[9] Eduardo DS, Franco SB, Castro JDV (2018). Magnetic resonance imaging of sellar and juxtasellar
abnormalities: atypical findings of common diseases and typical findings of
rare diseases. Radiol Bras.

